# Comparative Investigation of Phosphate Adsorption Efficiencies of MOF-76 (Ce) and Metal Oxides Derived from MOF-76 (Ce)

**DOI:** 10.1021/acs.langmuir.3c03369

**Published:** 2024-02-19

**Authors:** Ferda Civan Çavuşoğlu, Gülsüm Özçelik, Şahika Sena Bayazit

**Affiliations:** † Chemical Engineering Department, Faculty of Engineering & Architecture, İstanbul Beykent University, Istanbul 34396, Türkiye; ‡ Institute of Nanotechnology and Biotechnology, Istanbul University-Cerrahpaşa, Istanbul 34500, Türkiye

## Abstract

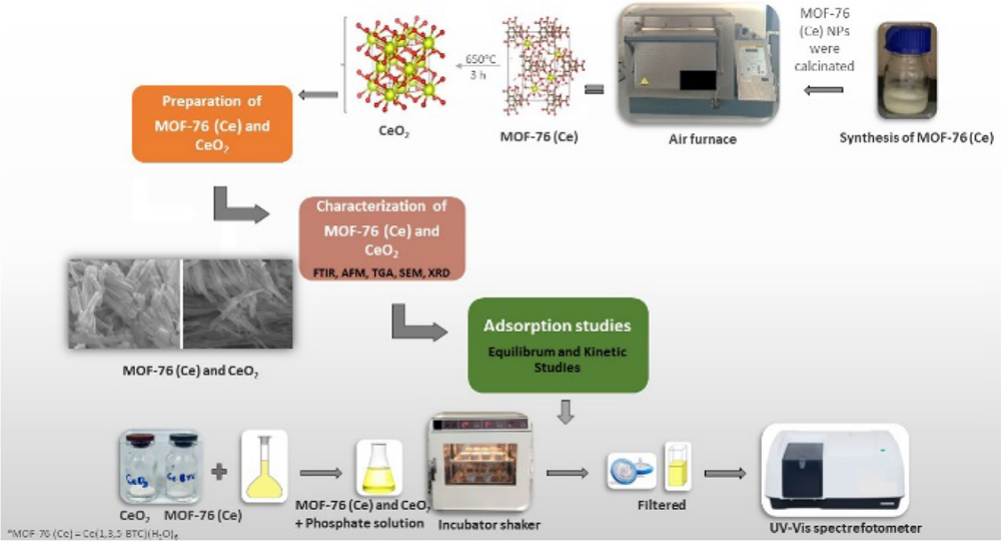

Phosphate pollution is a very challenging problem for the water environment. Phosphate mixed with water in various ways causes eutrophication. To sustain life in aquatic systems, phosphate ions must be cleaned. Therefore, it is very important to remove phosphate in wastewater. Here, an adsorption method has been tried for the removal of phosphate. MOF-76 (Ce), a cerium-based metal–organic framework, was synthesized by a hydrothermal method. Since metal oxides are known to be successful in phosphate adsorption, CeO_2_ nanoparticles were also obtained by pyrolysis of this MOF structure. The phosphate adsorption efficiencies of both adsorbents were compared. The characterization methods (SEM, FTIR, XRD, and TGA) were applied to adsorbents. The kinetic, isotherm, and thermodynamics studies were applied to experimental results. At 298 K, the adsorption capacity of MOF-76 (Ce) is higher than that of CeO_2_, according to Langmuir isotherm *q*_m_ values. The *q*_m_ values are 72.97 and 55.71 mg/g, respectively. Both adsorbents follow the pseudo second-order kinetic model. It has been found that MOF-76 (Ce) has a pH-selective property in phosphate adsorption. No change was observed in the phosphate adsorption capacity of CeO_2_ with pH. In terms of thermodynamics, the endothermic reaction is valid for both adsorbents.

## Introduction

Water pollution, which is largely due to human activity, is increasing day by day. The amount of wastewater is increasing due to problems such as developing industries, rapid urbanization, and population growth.^1^ Today, with the increase in consumption, the need for clean water is increasing at the same rate. In this context, reuse of water with treatment is of great importance.^2^ The rapid depletion of water reserves and the associated drought have enabled the reuse of wastewater to gain momentum.

Phosphorus is one of the essential elements of the human body. However, its excess creates a big problem for the environment by damaging water quality and the natural ecosystem. It is found in water in the form of phosphorus, orthophosphate (inorganic phosphate), organic phosphate, and polyphosphate. Phosphorus in wastewater is in the form of orthophosphate, except that it is present as a small amount of organic phosphate.^3,4^ Phosphates are discharged into water because of agricultural activities and domestic and industrial wastes. As a result of the accumulation of phosphates in the water by sewage discharges from fertilizers and detergents, the natural ecology of the water is disrupted. If this situation, which poses a great threat to aquatic creatures and the environment, cannot be prevented, water quality will deteriorate.^5,6^

When the phosphate concentration rises above a certain level, it causes eutrophication in lakes and rivers. With the increased algae formation because of eutrophication, the creatures living in lakes and rivers cannot get enough sunlight and oxygen. This eventually results in the death of aquatic life. For this reason, solutions have been sought to reduce the phosphate concentration in water.^7,8^ Some countries have limited the phosphate discharge, which is 0.5–1.0 mg of P/L in the USA and 5 mg of P/L in India.^9^ In addition, EPA states that according to water quality criteria, phosphates in water should not exceed 0.05 mg/L. The release of phosphate into surface waters is environmentally important, and phosphate removal from wastewater has been extensively studied.^10^ Removal of phosphates from wastewater has attracted great interest since the late 1960s to improve water quality. Phosphates are removed from water by basically three methods. These are physical, chemical, and biological methods.^11^ Among the physical methods, electrodialysis has a high capital cost, and reverse osmosis has a high energy cost. These methods have proven to be insufficient, removing only 10% of the total phosphorus from water. Chemical precipitation is a widely used method. However, it is not preferred due to its low efficiency, excessive use of chemicals, the costs of storage and feeding systems of these chemicals, and finally, large volume of waste sludge production. Biological methods are also not preferred because of their low efficiency, infrastructure investment cost, and uncontrolled microbial growth.^12,13^ Among these methods, adsorption is considered one of the promising technologies in phosphate removal. Adsorption is simple to use, low cost, and a more useful method and provides phosphate removal on high efficiency.^14^ For this purpose, many adsorbents such as fly ash,^15−17^ steel slag,^18,19^ red mud,^20^ zirconium,^21,22^ and iron-based components^23−25^ have been widely used to remove phosphate from aqueous media. Since these adsorbents have low phosphate removal efficiency, new adsorbents have begun to be developed.^26^

Metal oxides are used efficiently for phosphate removal. Acelas et al. prepared hydrated metal oxides dispersed anionic exchange media.^27^ Li et al. used iron(III)-copper(II) binary oxides for phosphate removal.^28^ Du et al. also synthesized La–Zr binary oxides for phosphate adsorption^29^ MOFs are also studied for phosphate removal. Commonly, Zr-based MOFs have been preferred for phosphate adsorption. Also, there have been some examples with Zn, Al, Fe, and Cu.^30^ In this study, MOF-76 (Ce) was prepared as a metal–organic framework. It was also used as the sacrificial template for metal oxide (CeO_2_) preparation. The adsorption efficiencies of crystal types of both MOF and metal oxides are investigated. Metal-containing adsorbents have been preferred due to their high affinity for the adsorption of anions, such as arsenate and phosphate. In this study, MOFs with cerium at their center and CeO_2_ derived from these MOFs were selected. The reason for choosing these materials is the high bonding capacity of cerium with phosphate. It has been stated in the literature that successful phosphate adsorption results were obtained with cerium compounds with different structures. In the study, it was tried to determine whether cerium would adsorb phosphate more efficiently in a MOF crystal structure or in the form of a metal oxide. The effects of the amount of adsorbent, solution pH, and coexisting ions are determined. The isotherm models, kinetic models, and thermodynamic analyses are applied to the experimental data.

## Materials and Method

### Materials

1,3,5-Benzene tricarboxylic acid (95%) and cerium nitrate hexahydrate (99%) were obtained from Sigma-Aldrich. Ethanol (99%), sodium chloride (>99%), hydrochloric acid (37%), sodium hydroxide (>98%), and potassium dihydrogen phosphate (>99%) were purchased from Merck Co.

### Preparation Methods of the MOF-76 (Ce) and CeO_2_

MOF-76 (Ce) [Ce(1,3,5-BTC)(H_2_O)_6_] nanoparticles were prepared by following the hydrothermal method. The cerium nitrate and 1,3,5-BTC were mixed equimolarly. The magnetic stirrer was used for this purpose. The solution was prepared in a water:ethanol (1:1) mixture. The reaction was carried out on 1 h, at 60 °C. After the reaction, white precipitate was removed from the reaction media by filtration. The obtained crystals were washed with water and ethanol, respectively. The drying process occurred at 60 °C overnight.^31−33^

The calcination process was carried out in an air furnace at 650 °C for 3 h.

### Surface Characterization Methods of the Adsorbents

The surface characterization of prepared adsorbents was occurred by using X-ray diffraction spectroscopy (XRD), thermal gravimetric analysis (TGA), scanning electron microscopy (SEM), and Fourier transform infrared spectroscopy (FTIR).

XRD analyses were performed by a Rigaku D/Max-2200 diffractometer (Cu Kα radiation with λ = 0.15418 nm). The XRD patterns were obtained between 10 and 60° with a 2°/min scanning speed. SEM images were acquired by FE-SEM, FEI Quanta FEG 450 at 30 kV. FTIR analyses were carried out by the KBr method (Bruker Alpha), and the wavenumber was adjusted between 400 and 4000 cm^–1^. After the adsorption processes, the FTIR analyses were repeated. TGA analyses were performed on a Hitachi STA-7200; the measurements were carried out between 20 and 800 °C under a nitrogen atmosphere; and the heating rate was chosen as 10 °C/min.

The point of zero charge analysis were applied to the adsorbents. The pH drift method was performed in this study. 0.01 M of NaCl solution was prepared, and then the pH of this solution was adjusted to 2, 4, 6, 8, 10, and 12 by using 0.1 M NaOH and 0.1 M HCl solutions. One mg of adsorbent was added to 20 mL of NaCl solutions at different pH values. The solid–liquid mixtures were shaken for 24 h at room temperature. After the reaction completed, the final pH values of supernatant NaCl solution were measured by a pH meter (WTW pH-meter). The diagonal intersection of the initial pH versus final pH plot gave the pH_pzc_.^34^

### Phosphate Adsorption Experiments

The batch type adsorption process was applied in this study. The phosphate adsorption on MOF-76 (Ce) and CeO_2_ nanoparticles was investigated in accordance with the effect of different parameters. These parameters were selected according to the literature. The adsorbent quantity, the solution pH, the co-ion concentration, temperature, and the initial phosphate concentration were chosen as adsorption parameters. The phosphate solution concentration range was chosen as 5–25 mg/L for isotherm experiments. The phosphate concentration was chosen as 20 mg/L for the kinetic, pH, co-ion effect, and adsorbent amount experiments. The general pH value of the adsorption system was about 5.5. The pH effect studies were applied at 3, 7, 9, and 11. 0.1 M NaOH (0.1 M) and HCl (0.1 M) were used for adjusting the acidity and basicity of the solution. The NaCl was chosen for determining the co-ion effect on phosphate adsorption. The NaCl concentration was adjusted between 0.005 and 0.1 M.

The phosphate concentration was determined by UV–visible spectrophotometry (Jasco V-730, Japan). The analysis was applied according to the vanadate–molybdate method at 410 nm.^35^ The adsorption uptake was calculated as follows
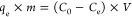
1*q*_e_ (mg/g) is the amount of adsorbed species on 1 g of adsorbent. *m* (g) is the adsorbent quantity. *C*_0_ (mg/L) is the initial phosphate concentration, and *C*_e_ (mg/L) is the equilibrium concentration of the phosphate. *V* (L) is the volume of the phosphate solution.

The adsorption kinetic model equations and equilibrium isotherm equations were applied to the experimental data.

The determination of the adsorption mechanism between adsorbent and adsorbate, four kinetic models were applied to experimental data. The pseudo first-order (PFO) kinetic model, pseudo second-order (PSO) kinetic model, Elovich model, and intraparticle diffusion model were chosen as basic modeling equations.

The nonlinear pseudo first-order kinetic model^36^ equation is given below,
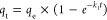
2

In this equation, *q*_e_ and *q*_t_ (mg/g) correspond to adsorption uptake at equilibrium and any t time. The rate constant is also shown as *k*_1_ (1/min).

The nonlinear pseudo second-order (PSO)^37^ kinetic model was also applied to experimental data. The PSO kinetic model equation is given as follows
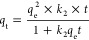
3

The *q*_e_ and *q*_t_ parameters represent the same values as those of the previous model. The PSO rate constant is indicated as *k*_2_ (g/mg min).

The Elovich model was applied as the third model to elucidate the kinetic mechanism of the adsorption system. The following equation is the Elovich kinetic model^38^
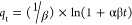
4

In eq 4, α is the initial adsorption rate (mg/g min) and β (g/mg) is constant related to the extent of surface coverage and activation energy for chemisorption (g/mg).

The latest model used to explain the phosphate adsorption mechanism is the intraparticle diffusion model.^39,40^ The equation of the intraparticle diffusion model is given below
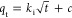
5

In eq 5, *k*_i_ (mg/g min^1/2^) is the intraparticle diffusion rate constant. *c* is the constant.

The adsorption isotherms are used for explaining the equilibrium behaviors of adsorbate–adsorbent systems. Langmuir, Freundlich, Temkin, and Dubinin–Radushkevich isotherms are chosen for phosphate adsorption on MOF-76 (Ce) and CeO_2_ nanoparticles. Whole isotherms were calculated as nonlinear type. The Langmuir isotherm assumes that the maximum adsorbate uptake that can be adsorbed onto the surface is equal to a single layer of molecules, with no interactions between the adsorbed molecules. The nonlinear Langmuir isotherm equation^41^ is as follows
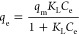
6

In eq 6, *q*_e_ and *q*_m_ (milligrams per gram) are the phosphate adsorption uptake at equilibrium and the calculated maximum phosphate adsorption uptake, respectively. *K*_L_ (L/mg) is the Langmuir constant. *K*_L_ is related to the adsorption enthalpy. *C*_e_ (mg/L) is the equilibrium concentration.

The Freundlich isotherm^42^ describes the multilayer adsorption on heterogeneous adsorption sites. The Freundlich isotherm equation is given below. In eq 7, *K*_F_ is the Freundlich constant, and 1/nis the adsorption intensity.
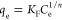
7

The Temkin isotherm^43^ gives information about the adsorption heat. According to this isotherm, increasing the occupied sites on the layer, the adsorption heat decreases linearly.^44^ The Temkin isotherm equation is given below
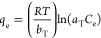
8

In eq 8, *R* (J/mol K) is the gas constant, *T* (K) is the temperature, *b*_T_ (J/mol) is the adsorption heat constant, and *a*_T_ (L/mg) is the equilibrium binding constant.

The Dubinin–Radushkevich (D-R) isotherm^45^ considers of the pores structure of the adsorbent. The isotherm equation is presented below
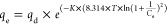
9
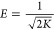
10

In eq 9, *K* is the D-R (mol^2^/kJ^2^) constant, *q*_d_ (mg/g) is the maximum adsorption capacity, and *E* (kJ/mol) is the mean adsorption energy. The calculation of *E* is given in eq 10.

## Results and Discussion

### Surface Characterization Analysis of the Adsorbents

**Figure 1:**
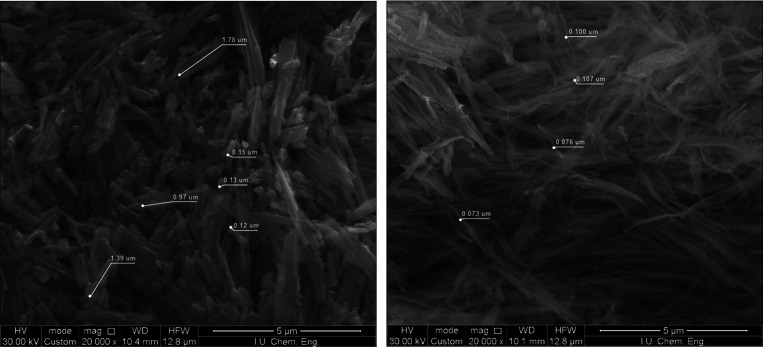
SEM images of MOF-76 (Ce) (left) and CeO_2_ (right).

The surface morphologies of the adsorbents were determined by SEM images. The SEM images of MOF-76 (Ce) and CeO_2_ are given in Figure 1. The crystal structures of the MOF-76 (Ce) nanoparticles are nanorod types. The nanorods can be observed clearly in Figure 1. With the collapse of the carbon skeleton structure after the heat treatment, the appearance of the nanorods changed to nanofibers. The length of MOF-76 (Ce) nanorods was measured as about 1–1.78 μm, and the width of the nanorods was measured as 0.12–0.15 μm. The effect of thermal treatment can be observed at the width of the CeO_2_ nanofibers. The width of the nanofibers was measured as about 0.07–0.10 μm. (Size analysis of SEM images were determined with ImageJ software.^46^) The nanorod structures consist of multiple smaller crystals. The rods are made of a brick-upon-tile pattern.^31^

**Figure 2:**
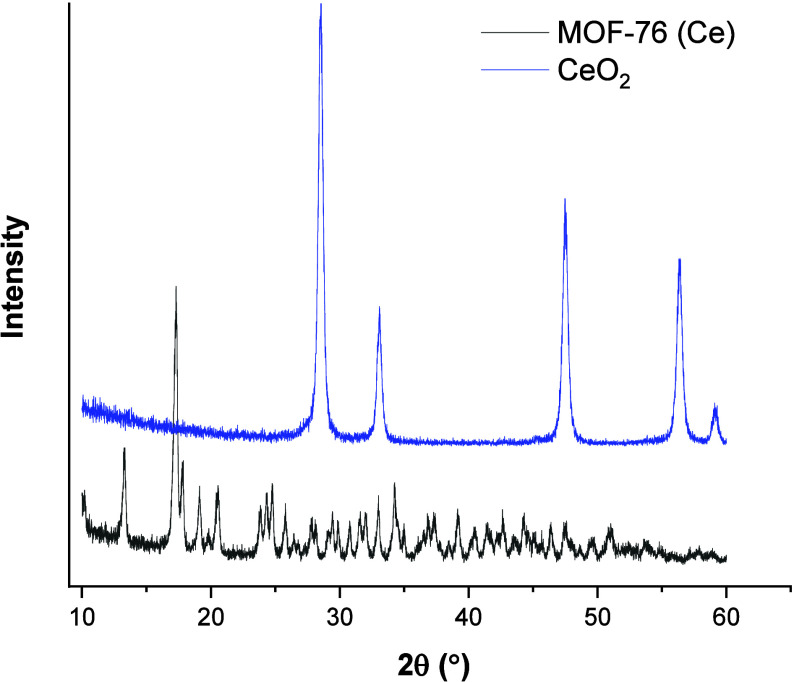
XRD patterns of MOF-76 (Ce) and CeO_2_ nanoparticles.

XRD patterns of MOF-76 (Ce) and CeO_2_ nanoparticles were given in Figure 2. The XRD peaks of MOF-76 (Ce) are compatible with the literature.^31^ The peaks are observed at 13.3, 17.3, 17.83, 19.2, and 20.4°. CeO_2_ peaks are observed at 28.5, 33.2, 47.5, 56.3, and 59.1° (JCPDS pattern no. 75–0076). The fwhm values of the peaks for both adsorbents were calculated by Gaussian function fitting. Then, the crystallite sizes of MOF-76 (Ce) and CeO_2_ were calculated by the Debye–Scherrer method. The Debye–Scherrer equation is *D* = *K*λ/βcosθ. *D* is the crystallite size, *K* is the Scherrer constant (0.98), λ represents the wavelength (1.54), and β is the full width at half-maximum (fwhm). The calculated average particle sizes of MOF-76 (Ce) and CeO_2_ are 23.63 and 18.36 nm, respectively.

**Figure 3:**
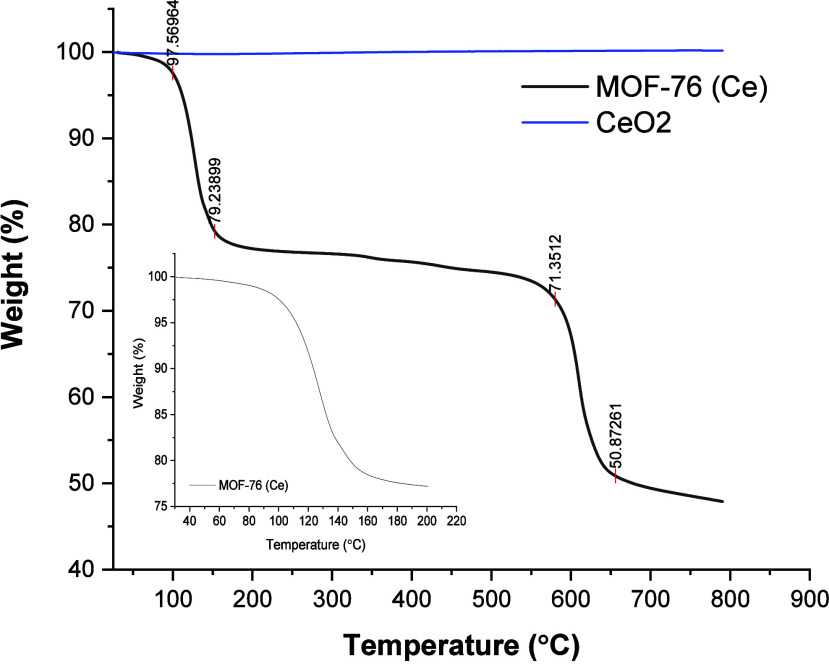
TGA plots of MOF-76 (Ce) and CeO_2_ nanoparticles; TGA plot of MOF-76 (Ce) between 30 and 200 °C.

Thermogravimetric analyses of MOF-76 (Ce) and CeO_2_ are illustrated in Figure 3. With the removal of moisture, an ∼2.5% decrease in mass was observed. The weight loss at 200 °C corresponds to H_2_O and DMF molecules inside the pores, and the weight loss ratio is about 18% for MOF-76 (Ce) nanoparticles. The weight loss for H_2_O and DMF molecules can be observed clearly at the inner plot in Figure 3. The highest rate of weight loss is the value where the temperature is high (550–600 °C). It is known that the organic linker is decomposed in this section. After the calcination of MOF-76 (Ce), CeO_2_ nanofibers were obtained. So, no difference was observed in the TGA plot of CeO_2_ nanofibers.

**Figure 4:**
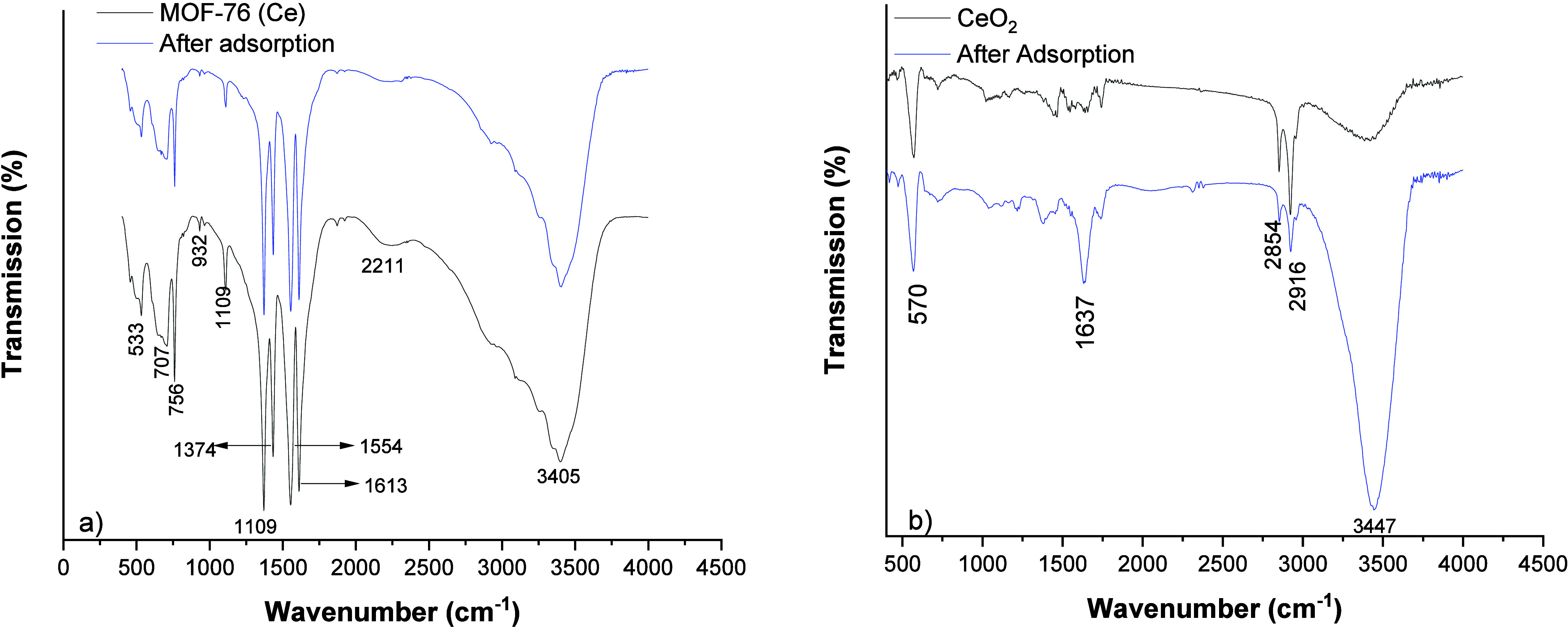
FTIR plots of MOF-76 (Ce) (a) and CeO_2_ (b) before and after phosphate adsorption.

FTIR plots of MOF-76 (Ce) and CeO_2_ are given in Figure 4a,b. In addition, FTIR plots after phosphate adsorption are shown in Figure 4a,b. The peaks in the FTIR spectrum of MOF-76 (Ce) at 1619–1554 cm^–1^ and 1435–1381 cm^–1^ are due to the stretching vibrations of asymmetric and symmetric carboxylate anions, respectively. The peak at 524 cm^–1^ is due to the Ce–O single bond vibrations. The peak at 3409 cm^–1^ is due to water used as a solvent. The Ce–O single bond vibration peak in the FTIR graph of CeO_2_ is located at 562 cm^–1^. The single bond OH peak is located at 3426 cm^–1^. The intensity of the carboxylate peaks decreased after calcination.

### Phosphate Removal Studies

#### Investigation of Adsorbent Quantity and Co-Ions Effects

**Figure 5:**
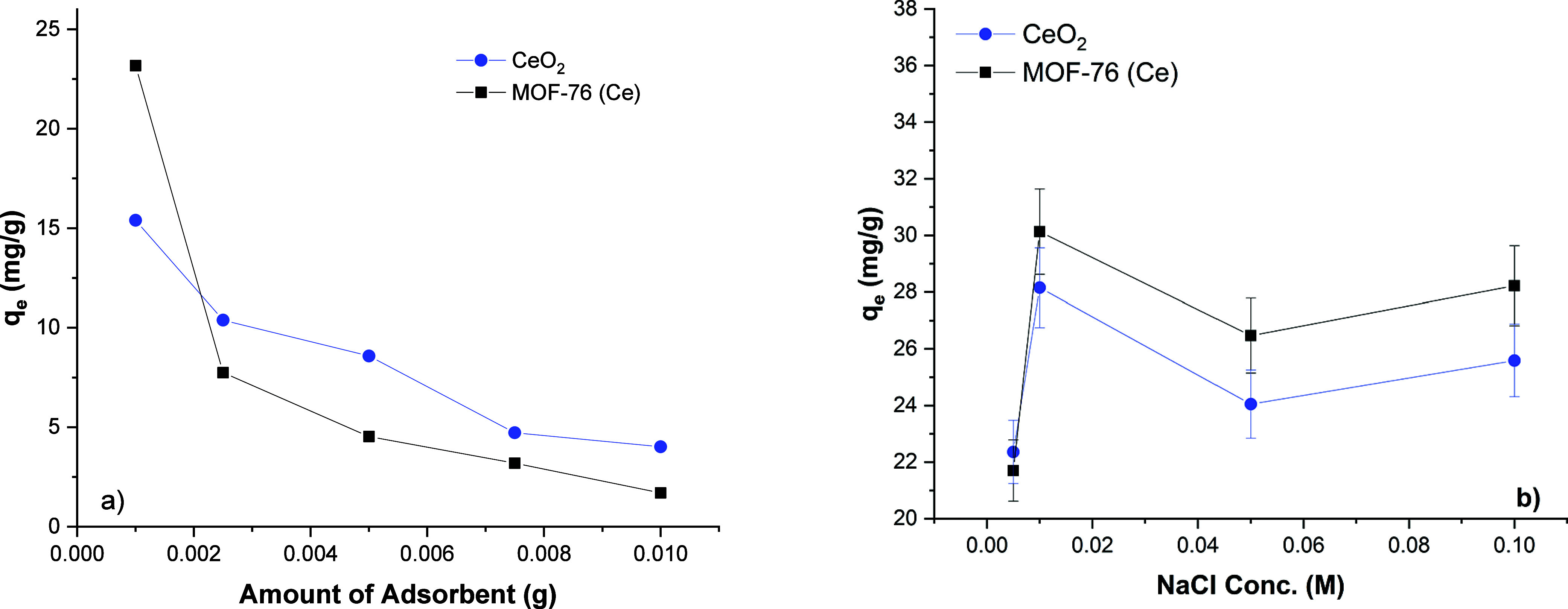
Effect of adsorbent quantities on phosphate adsorption of MOF-76 (Ce) and CeO_2_ (a); co-ion effect on phosphate adsorption of MOF-76 (Ce) and CeO_2_ (b).

The adsorbent quantity is one of the most important variables in optimizing the adsorption process. The chosen quantities of adsorbents are between 0.1 and 1 g/L. The initial phosphate ion concentration was adjusted as 20 mg/L. The effect of adsorbent quantities is illustrated as a plot in Figure 5a. It can be understood from the plot that for a more efficient adsorption process, lower adsorbent quantities are more suitable. Also, it is understood that MOF-76 (Ce) is a more effective adsorbent for low adsorbent quantities with a significant difference compared to CeO_2_. As depicted in Figure 5a, when 1 mg of adsorbent is used, the adsorption capacity of MOF-76 (Ce) reaches 23.17 mg/g. The adsorption capacity of CeO_2_ remained around 15.39 mg/g. It is understood that CeO_2_ gives more successful results as the amount of adsorbent increases. However, for an economically sustainable adsorption process, the optimum amount of adsorbent was chosen as 1 mg in this study.

The NaCl was chosen as the co-ion for determining the effects of different molecules on phosphate adsorption. The results are given in Figure 5b. The initial phosphate concentration is 20 mg/L, and different concentrations of Cl^–^ are added to the solutions. The determined NaCl concentrations are between 0.01 and 0.1 M. The CeO_2_ adsorbent is less affected by the addition of chloride ion to the medium. When Figure 5b is examined, it is understood that the phosphate adsorption uptake is 22–28 mg/g when the chloride ion concentration is changed between 0.01 and 0.1 M. Moreover, it is noticed in Figure 5b that the interaction of MOF-76 (Ce) adsorbent with chloride ion shows the same trend. It is observed that the adsorption uptake values of MOF-76 (Ce) swing between 22 and 30 mg/g. Outer sphere complexation and inner sphere complexation may occur during the adsorption of anions by metal oxides or metal-containing adsorbents. It has been stated in the literature that the system is very sensitive to ionic strength in case of outer sphere complexation. It has been observed that the complex structure formed during inner sphere complexation has low sensitivity to ionic resistance.^47^ It has been reported that chloride ions facilitate chelation with H_2_PO_4_^–^ in their intermediate complexes with metal oxides.^48^

#### Effect of Contact Time and Elucidation of Adsorption Kinetics

**Figure 6:**
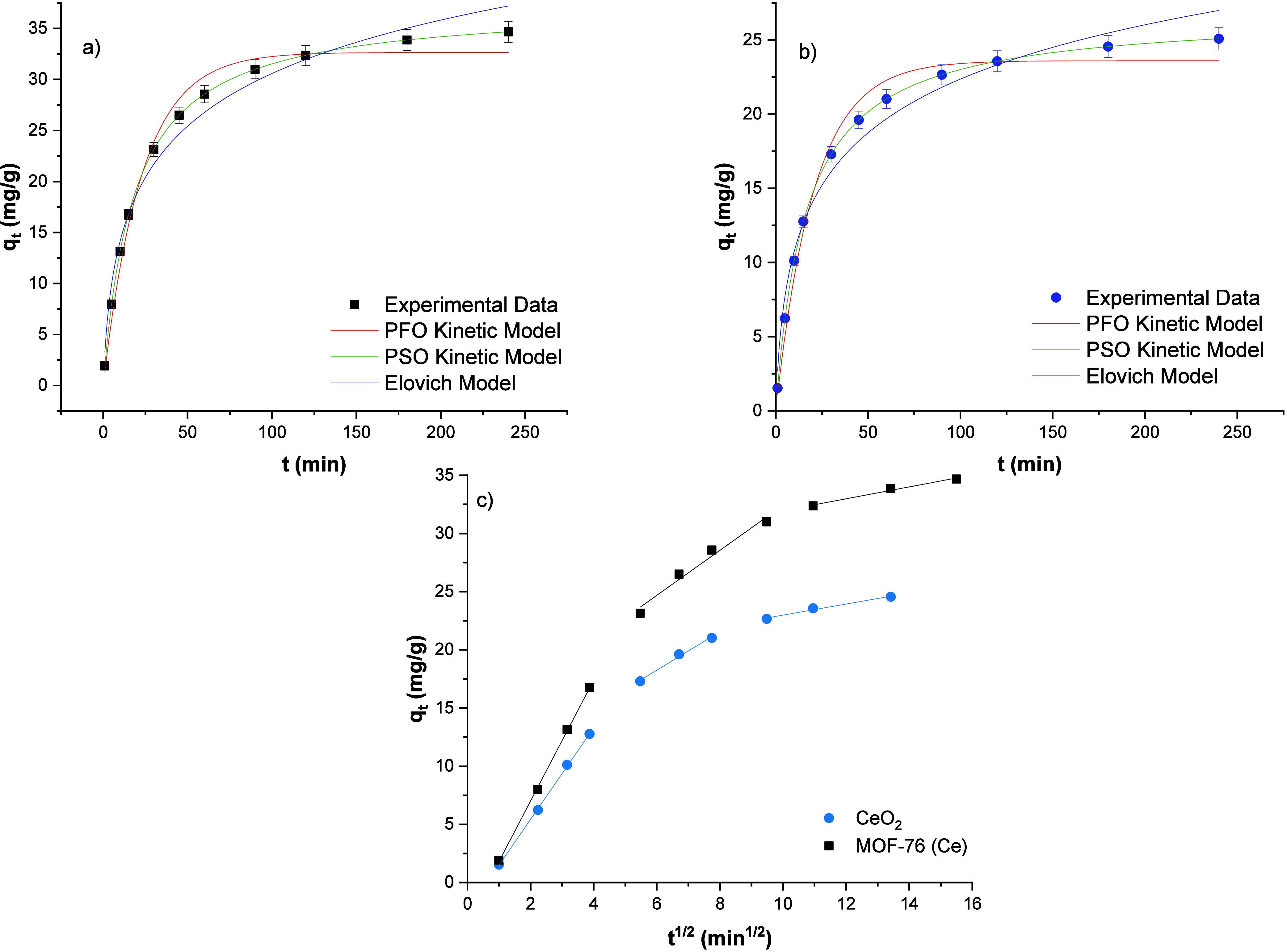
Phosphate adsorption kinetic plots of MOF-76 (Ce) (a) and CeO_2_ (b); intraparticle diffusion model for both adsorbents (c).

The equilibrium periods of phosphate adsorption on MOF-76 (Ce) and CeO_2_ nanoparticles were determined by contact time experiments. As observed in Figure 6a,b, equilibrium time for the phosphate adsorption process on both adsorbent is approximately 150–180 min. In this study, 180 min was chosen as the equilibration time. The agitation speed was chosen as 120 rpm. The agitation speed is important for decreasing of mass transfer resistance between the surface and phosphate ions.^49^ 120 rpm was used as a constant and sufficiently high agitating speed.

The nonlinear PFO, PSO, and Elovich kinetic plots are given in Figure 6a,b. The linear intraparticle diffusion plots are given in Figure 6c. Also, the calculated parameters are shown in Table 1. When the correlation coefficients are examined, it is understood that the phosphate adsorption kinetic mechanism follows the general model equations. Considering the parameters of the PFO and PSO kinetic models, the experimental *q*_e_ values and the calculated *q*_e_ values are very close to each other, so both kinetic models can be helpful in expressing the phosphate adsorption mechanism on the MOF-76 (Ce) and CeO_2_ nanoparticles. In addition, the *R*^2^ values of the PFO model are 0.98 and the *R*^2^ values of the PSO model are 0.99. However, as observed in Figure 6a,b, the PSO kinetic model fits better than the PFO and Elovich models. The chi-square test was applied for determining the best fitted kinetic model. As observed in Table 1, the PSO kinetic model has the lowest chi-square values for both adsorbents. Therefore, it is appropriate to say that the PSO model is the most suitable model to describe the phosphate adsorption mechanism of both the MOF-76 (Ce) and CeO_2_ nanoparticles. The rate limiting step of PSO kinetic model is about the collision between the nonadsorbed adsorption sites. The adsorption uptake decreases over time, because of diffusion limited the pore filling.^50^ The calculated adsorption uptakes of MOF-76 (Ce) and CeO_2_ are 37.33 and 26.81 mg/g, respectively. The adsorption uptakes of PFO kinetic model are 32.66 and 23.61 mg/g, respectively. The rate limiting step of the PFO model is collisions between adsorbate molecules with nonadsorbed sites at the surface of the MOF-76 (Ce) and CeO_2_. The Elovich model shows that the initial adsorption rates of MOF-76 (Ce) and CeO_2_ are very high according to general adsorption rates calculated by PFO and PSO models. The α values of MOF-76 (Ce) and CeO_2_ are 4.09 and 3.46 mg/g min, respectively. The *k*_2_ values are 0.0015 and 0.0023 min^–1^, respectively. The intraparticle diffusion model plots generally consist of 2 or 3 steps. Each step shows different stages of phosphate adsorption. For the phosphate adsorption on MOF-76 (Ce) and CeO_2_ nanoparticles, the process occurs three stages as observed in Figure 6c. These three stages are the initial curved, linear, and plateau sections. The first stage is the boundary layer diffusion step, and this step occurs rapidly. The *k*_i_ rate constants of MOF-76 (Ce) and CeO_2_ are 5.19 and 3.93 mg/(g min^1/2^), respectively. The second stage is the intraparticle diffusion step, and this stage is the rate-controlling section. The rate constants decrease sharply at this stage. The last stage is the equilibrium step, and the adsorption rate is declining considerably.^51^ According to elucidation of the kinetic mechanisms of phosphate adsorption on MOF-76 (Ce) and CeO_2_, MOF-76 (Ce) has a higher phosphate adsorption capacity, and the adsorption process takes place faster. There are more than one rate-controlling steps, and these are chemisorption due to the PSO kinetic model and intraparticle diffusion.

**Table 1:** Calculated Kinetic Model Parameters of Phosphate Adsorption on MOF-76 (Ce) and CeO_2_

		MOF-76 (Ce)			CeO_2_		
experimental *q*_e_		34.67			25.04		
pseudo first-order kinetic model	*q*_e_ (mg/g)	32.66			23.61		
	*k*_1_ (min^–1^)	0.044			0.048		
	*R*^2^	0.98			0.98		
	Χ^2^	2.11			1.16		
pseudo second-order kinetic model	*q*_e_ (mg/g)	37.33			26.81		
	*k*_2_ (g/mg min)	0.0015			0.0023		
	*R*^2^	0.99			0.99		
	Χ^2^	9.18 × 10^–28^			5.32 × 10^–28^		
Elovich kinetic model	α (mg/g min)	4.09			3.46		
	β (g/mg)	0.13			0.18		
	*R*^2^	0.98			0.97		
	Χ^2^	2.76			1.59		
intraparticle diffusion model	*k*_i_ (mg/g min^1/2^)	5.19	1.93	0.51	3.93	1.65	0.48
	*c*	–3.39	13.08	26.84	–2.45	8.34	18.22
	*R*^2^	0.99	0.96	0.97	0.99	0.98	0.97

#### Elucidation of Adsorption Equilibrium Isotherms and Adsorption Mechanisms

**Figure 7:**
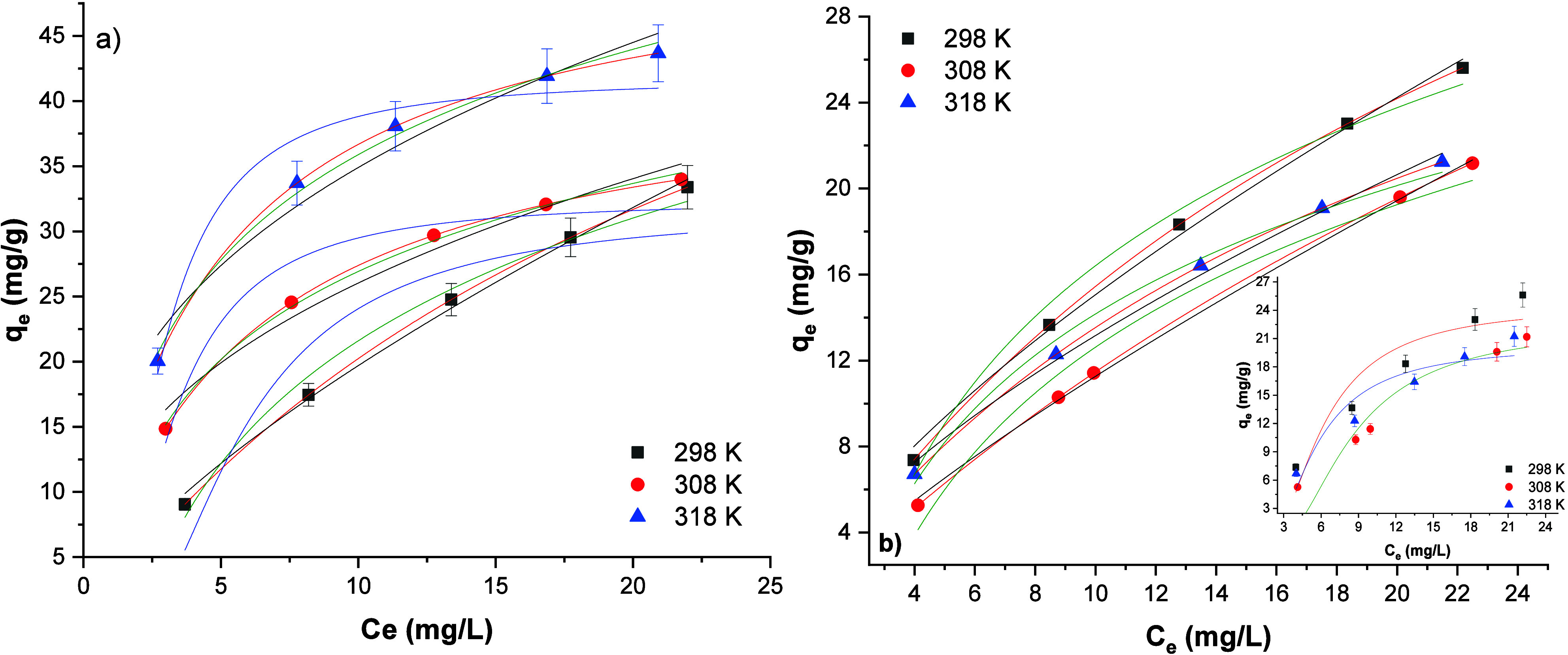
Phosphate adsorption equilibrium plots at different temperatures of MOF-76 (Ce) (a) (Langmuir isotherm: red line; Freundlich isotherm: black line; Temkin isotherm: green line; Dubinin–Radushkevich isotherm: blue line), and CeO_2_ (b) (Langmuir isotherm: red line; Freundlich isotherm: black line; Temkin isotherm: green line; Dubinin–Radushkevich isotherm: inner plot).

Langmuir, Freundlich, Temkin, and Dubinin–Radushkevich isotherms are chosen for phosphate adsorption on MOF-76 (Ce) and CeO_2_ nanoparticles. The isotherm plots are given in Figure 7a,b. Also, the calculated parameters are given in Table 2. Three different temperatures were tested for adsorption isotherms. These temperatures are 298, 308, and 318 K. As can be seen from Table 2 and Figure 7, phosphate adsorption on MOF-76 (Ce) and CeO_2_ nanoparticles follows the Langmuir isotherm, clearly. The *R*^2^ values of both adsorbents at three different temperatures are 0.99. Also, the *R*^2^ values of the Freundlich isotherm are above the acceptable limit (0.96–0.99). Therefore, it can be said that the adsorption of phosphate on MOF-76 (Ce) and CeO_2_ nanoparticles fits both isotherms. The maximum phosphate adsorption capacity, determined by the *q*_m_ value from Langmuir isotherm parameters, was found to be 72.97 mg/g of MOF-76 (Ce) at 298 K and 65.61 mg/g of CeO_2_ at 308 K. The initial phosphate concentrations are between 5 and 25 mg/L. He et al., prepared Ce (III) nanocomposites for phosphate adsorption, the initial phosphate concentration range is 50–500 mg/L, and the calculated *q*_m_ values are 189.4 mg/g.^52^ Mohammadi et al. prepared biochar from *Rosmarinus officinalis* leaves and ZnO for phosphate removal. The maximum adsorption capacities were 50.47 and 35.95 mg/g.^53^ Lin et al. used UiO-66 and UiO-66-NH_2_ MOFs for phosphate adsorption. The initial phosphate concentration range was chosen as 5–100 mg/L. The obtained *q*_m_ values are 85 mg/g for UiO-66 and 92 mg/g for UiO-66-NH_2_. Different adsorbents used for phosphate adsorption in the literature and the adsorption uptakes obtained from them are listed in Table 3. Langmuir isotherm advocates the mechanism of monolayer adsorption of phosphate ions to MOF-76 (Ce) and CeO_2_ nanoparticles. Freundlich isotherm describes the heterogeneity of surfaces and multilayer adsorption. In addition, the 1/*n* parameter of the Freundlich isotherm gives information about the interaction between the adsorbent and adsorbate. If the value of 1/*n* is between 0 and 1, the adsorption process is favorable and indicates a chemical interaction between phosphate and solid surfaces. According to Table 2 data, it is understood that both adsorbents also follow the Temkin isotherm in the adsorption of phosphate ions. The Temkin isotherm is a suitable model for systems where chemical adsorption by electrostatic attraction of positive and negative charges is valid.^54^ Therefore, for both adsorbents, it can be thought that there is a system that starts with electrochemical attraction and ends with chemical adsorption. The *R*^2^ values of D-R isotherms of phosphate adsorption on MOF-76 (Ce) and CeO_2_ nanoparticles are under the 0.90 level. However, the *q*_d_ (maximum adsorption capacity) values are close to experimental results. According to mean adsorption energy values, the physical adsorption has occurred at 298 K for MOF-76 (Ce) and chemisorption has occurred at 308 and 318 K. This is because, if *E* > 8 kJ/mol, the adsorption mechanism is chemisorption and if *E* < 8 kJ/mol, the system follows physical adsorption. Due to the D-R model, the phosphate adsorption on CeO_2_ nanoparticles should follow physical adsorption because *E* values are between 4.56 and 6.76 kJ/mol. However, the D-R isotherm used to describe solid–liquid phase adsorption ignores the solvent effect and the effect of solution pH on the surface. It also ignores the surface charges of adsorbents and the dissociation of functional groups. The D-R isotherm cannot fully provide the mean adsorption energy to distinguish between a solid–liquid phase adsorption, physical, or chemical adsorption.^55^

**Table 2:** Calculated Isotherm Model Parameters of Phosphate Adsorption on MOF-76 (Ce) and CeO_2_

		MOF-76 (Ce)			CeO_2_		
		298 K	308 K	318 K	298 K	308 K	318 K
Langmuir	*q*_m_	72.97	42.77	52.93	55.71	65.61	42.02
	*K*_L_	0.038	0.178	0.226	0.038	0.021	0.048
	*R*^2^	0.99	0.99	0.99	0.99	0.99	0.99
Freundlich	*K*_f_	3.99	10.68	15.57	3.09	1.84	2.95
	1/*n*	0.69	0.38	0.35	0.68	0.78	0.65
	*R*^2^	0.99	0.97	0.96	0.99	0.99	0.99
Temkin	*b*_t_	182.25	262.68	226.60	233.55	306.70	267.65
	*a*_t_	0.49	1.58	2.17	0.47	0.52	0.37
	*R*^2^	0.98	0.99	0.99	0.98	0.99	0.98
D-R	*q*_d_	31.71	32.37	41.70	24.55	22.51	20.32
	*K*	0.012	0.004	0.003	0.013	0.024	0.011
	*E*	6.46	11.24	12.91	6.20	4.56	6.76
	*R*^2^	0.88	0.93	0.93	0.88	0.88	0.89

**Table 3:** Literature Summary of Different Kinds of Adsorbents Used for Phosphate Adsorption

adsorbent	solution pH	phosphate concentration (mg/L)	maximum uptake (mg/g)	isotherm model
Metal Oxides/Hydroxides				
granular ferric hydroxide^59^	7.2	1–100	20.9	Langmuir
FerroSorp Plus^59^			23.6	
compacted ferric (hydr)oxides^59^			26.6	
aluminum oxides^59^			20.9	
iron oxide doped halloysite nanotubes^47^	4	1–100	5.42	Langmuir
clay minerals				
Mg–Al hydrotalcite-loaded kaolin clay^60^	7.5	10–200	11.92	Langmuir
bentonite^61^	7	1–50	7.13	Langmuir
kaolinite^61^			5.37	
Modified Adsorbents				
iron pillared eggshell modified bentonite clay composite^62^	3	15–100	60.51	Langmuir
iron–carbon nanotube composites^63^		10–800	36.5	Langmuir
Mg coprecipitated peanut shell biochar^64^		5–100	50.58	Langmuir
lanthanum encapsulated chitosan-kaolin clay hybrid composite^65^	5.3	50–300	106.48	Langmuir
Biomaterials				
quaternary ammonium-based sugar cane bagasse^66^	5.0 ± 0.3	5–300	32.1	Langmuir
chitosan-modified sugar cane bagasse biochar^67^	3	5–100	37.21	Liu
scallop shell-based ceramic biomaterials^68^	5.7 ± 0.2	10–50	13.95	Langmuir
MOF-76 (Ce) (this study)	5.5	5–25	72.97	Langmuir
CeO_2_ (this study)			55.71	

**Figure 8:**
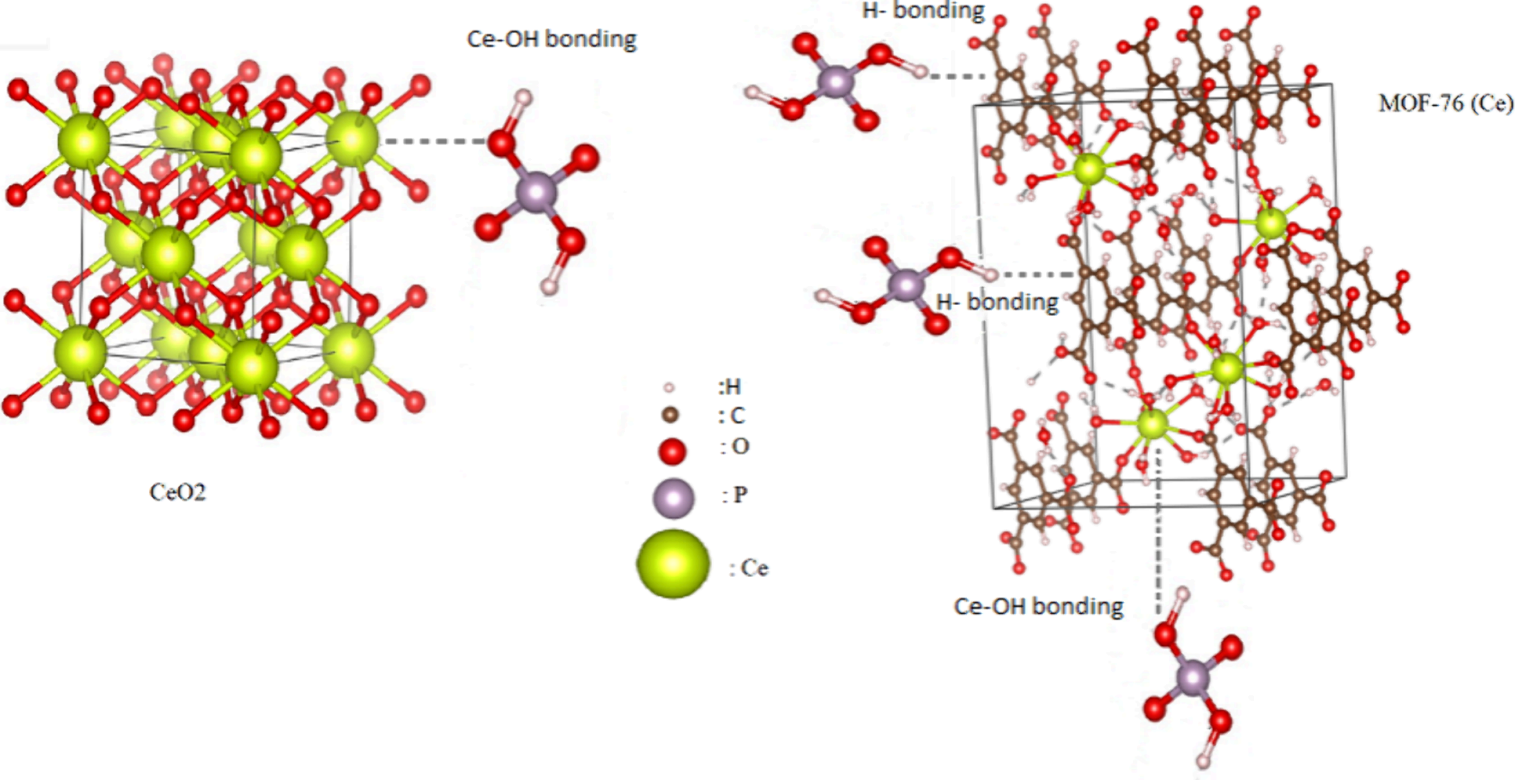
Scheme of the estimated phosphate adsorption mechanism on CeO_2_ and MOF-76 (Ce).

Postadsorption FTIR analyses were also performed to reveal the MOF-76 (Ce) and CeO_2_ adsorption mechanism of phosphate ions. In Figure 4a,b, the plots after adsorption are also depicted. There was no significant difference in the FTIR peaks after adsorption. The intensity of some peaks decreased after adsorption for the MOF-76 (Ce) nanoparticles. It is observed that the intensity of the C–O stretching peak at 1109 cm^–1^ and the C=C bending peak at 932 cm^–1^ decrease. MOF-76 (Ce) nanoparticles generally adsorb phosphate ions by forming hydrogen bonds. In Figure 8, the predicted adsorption mechanism is illustrated. As seen in the FTIR analysis, the intensity of the C–C bonds decreased. HPO_4_^2–^ and H_2_PO_4_^–^ are known to be hydrogen donors.^56^ Therefore, it is possible for hydrogen bonds to form between the MOF-76 (Ce) and phosphate ions. There are also strong interactions between Ce and O. For this reason, it is predicted that there will be an interaction with the Ce center and the oxygen and hydroxyl groups of the phosphate ions.^57^ When the FTIR analysis of CeO2 nanoparticles is examined, it is observed that the intensity of the −OH peak at 3447 cm^–1^ increased considerably after phosphate adsorption. This result also strengthens the prediction of interaction between the Ce center and the −OH ion of the phosphate ion, which is indicated in Figure 8. The molecular structures were drawn at Vesta 3 software.^58^

#### Effect of Solution pH on Adsorption

**Figure 9:**
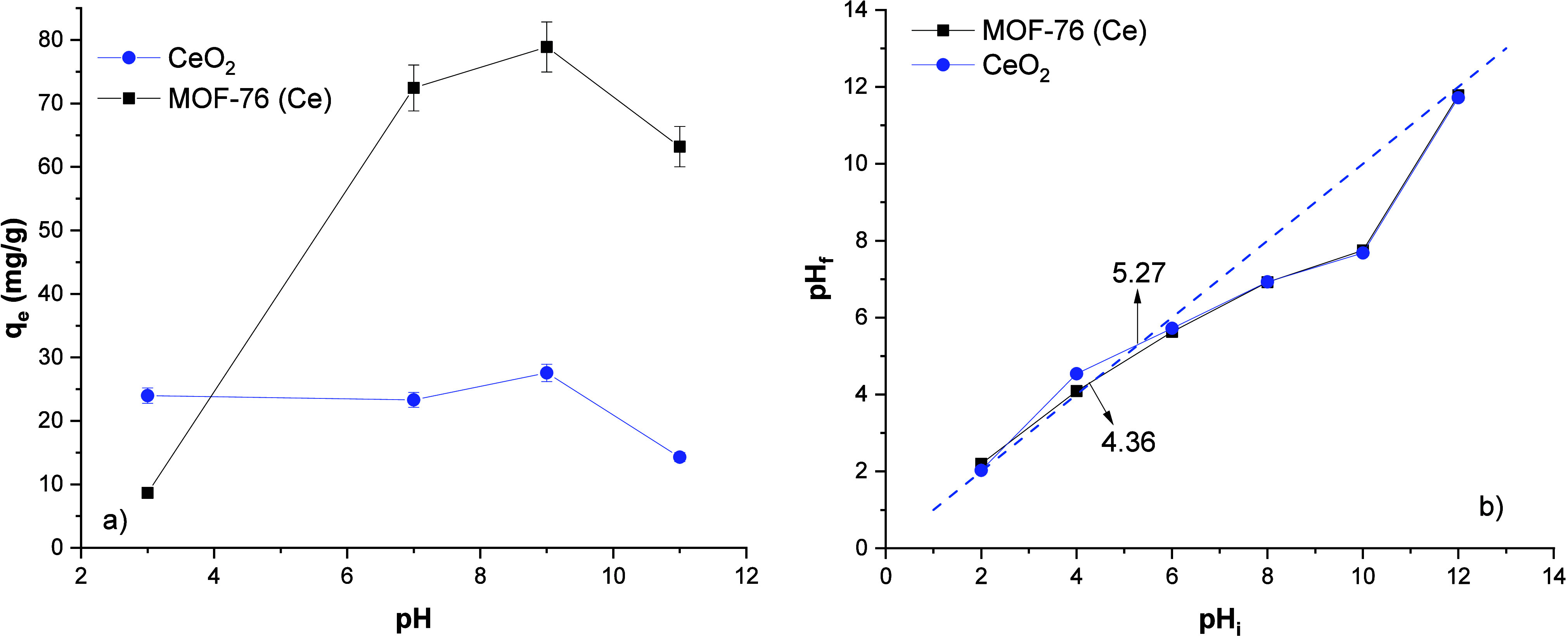
Effect of solution pH on phosphate adsorption of MOF-76 (Ce) and CeO_2_ (a), pH_zpc_ plots of MOF-76 (Ce) and CeO_2_ (b).

The pH effect plots of phosphate adsorption on the MOF-76 (Ce) and CeO_2_ nanoparticles are given in Figure 9a. The pH_zpc_ values of MOF-76 (Ce) and CeO_2_ nanoparticles were found as 4.36 and 5.27, respectively, as seen in Figure 9b. The distribution of phosphate ions at different pH values are as follows; H_3_PO_4_ pH < 2, H_2_PO_4_^–^ 3 < pH < 7, HPO_4_^2–^ 7 < pH < 11 and PO_4_^3–^ pH > 11. As seen in Figure 9a, the solution pH has no significant effect on phosphate adsorption for CeO_2_ nanoparticles. Since the phosphate ion reaches −3 valence after pH 11, it becomes difficult to interact with the surface. Phosphate adsorption on MOF-76 (Ce) increases in the pH range of 7–11. It is known that the surface is negatively charged at pH values above the pH_zpc_ value. In the case of MOF-76 (Ce), when pH > 6, Ce (III) ions in the structure are hydrolyzed and lower the solution pH by binding −OH ions in the environment.^52^ Therefore, the phosphate adsorption process behaves as an acidic media, and the adsorption efficiency is increased.

#### Thermodynamic Studies of Phosphate Adsorption on MOF-76 (Ce) and CeO_2_ Nanoparticles

Thermodynamic parameters were calculated by using van’t Hoff equation. The *K*_L_ values of the Langmuir isotherm were used as the equilibrium constant.
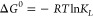
11

Gibbs free energy of the system was calculated according to eq 11. *R* is the gas constant, and *T* is temperature.
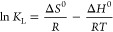
12

In eq 12, Δ*S*^0^ and Δ*H*^0^ are determined by the slope and the intercept of the linear plot between ln *K*_L_ and 1/*T*. The calculated parameters are given in Table 4. All Δ*G*^0^ values are negative so the phosphate adsorption on MOF-76 (Ce) and CeO_2_ nanoparticles is a spontaneous and favorable process. Δ*G*^0^ values decrease while temperature increases; therefore, the spontaneity is also increasing with temperature. The positive Δ*S*^0^ values show good affinity of phosphate ions to the surfaces of the adsorbents. Δ*H*^0^ values also show that the system is endothermic for both adsorbents. The positive effect of *T* rises on spontaneity with the endothermic system.

**Table 4:** Calculated Thermodynamics Parameters of Phosphate Adsorption on MOF-76 (Ce) and CeO_2_

	Δ*G*° (kJ/mol)		Δ*H*° (kJ/mol)	Δ*S*° (J/mol)
MOF-76 (Ce)	298 K	–14.69	70.76	288.44
	308 K	–19.14		
	318 K	–20.38		
CeO_2_	298 K	–14.69	8.59	76.22
	308 K	–13.66		
	318 K	–16.29		

## Conclusions

In this study, a Ce-based metal organic framework was synthesized, and CeO_2_ was produced by pyrolyzing of the MOF nanoparticles. According to SEM analysis, it was observed that MOF-76 (Ce) and CeO_2_ grew in the form of nanofibers. The general adsorption parameters were investigated in three sections: kinetics, equilibrium, and thermodynamics. Both adsorbents reach equilibrium in approximately 180 min. Also, the highest correlation coefficient was obtained in the pseudo-second-order kinetic model. Thus, the rate-limiting step of the reaction is chemisorption. Langmuir and Freundlich isotherms were the models that best explained the adsorption equilibrium. According to the Langmuir isotherm and adsorption uptake values in the kinetic models, the phosphate adsorption capacity of MOF-76 (Ce) is considerably higher than that of CeO_2_. This result is due to both the interaction between Ce–OH and the ability of MOF-76 (Ce) to form hydrogen bonds, as visualized in Figure 8, where the estimated adsorption mechanism is mentioned. The solution pH is an important parameter for MOF-76 (Ce) but is not significant for CeO_2_. MOF-76 (Ce) has selectivity toward to pH values of phosphate solution. This is a very important gain for the adsorption process. It is a thermodynamically spontaneous and favorable system. It can be said that phosphate adsorption is an endothermic reaction for both adsorbents.

## References

[ref1] Mulkerrins D.; Dobson A. D. W.; Colleran E. Parameters Affecting Biological Phosphate Removal from Wastewaters. Environ. Int. 2004, 30, 249–259. 10.1016/S0160-4120(03)00177-6. 14749113

[ref2] Peleka E. N.; Deliyanni E. A. Adsorptive Removal of Phosphates from Aqueous Solutions. Desalination 2009, 245 (1–3), 357–371. 10.1016/j.desal.2008.04.050.

[ref3] Oguz E. Removal of Phosphate from Aqueous Solution with Blast Furnace Slag. J. Hazard Mater. 2004, 114 (1–3), 131–137. 10.1016/j.jhazmat.2004.07.010. 15511583

[ref4] Peleka E. N.; Deliyanni E. A. Adsorptive Removal of Phosphates from Aqueous Solutions. Desalination 2009, 245 (1–3), 357–371. 10.1016/j.desal.2008.04.050.

[ref5] Loganathan P.; Vigneswaran S.; Kandasamy J.; Bolan N. S. Removal and Recovery of Phosphate From Water Using Sorption. Crit Rev. Environ. Sci. Technol. 2014, 44 (8), 847–907. 10.1080/10643389.2012.741311.

[ref6] Liu J.; Thallapally P. K.; Mcgrail B. P.; Brown D. R.; Liu J. Progress in Adsorption-Based CO 2 Capture by Metal-Organic Frameworks. Chem. Soc. Rev. 2012, 41, 2308–2322. 10.1039/C1CS15221A. 22143077

[ref7] Awual M. R.; Jyo A.; Ihara T.; Seko N.; Tamada M.; Lim K. T. Enhanced Trace Phosphate Removal from Water by Zirconium(IV) Loaded Fibrous Adsorbent. Water Res. 2011, 45 (15), 4592–4600. 10.1016/j.watres.2011.06.009. 21724222

[ref8] Wu B.; Wan J.; Zhang Y.; Pan B.; Lo I. M. C. Selective Phosphate Removal from Water and Wastewater Using Sorption: Process Fundamentals and Removal Mechanisms. Environ. Sci. Technol. 2020, 54, 50–66. 10.1021/acs.est.9b05569. 31804806

[ref9] Vasudevan S.; Sozhan G.; Ravichandran S.; Jayaraj J.; Lakshmi J.; Sheela S. M. Studies on the Removal of Phosphate from Drinking Water by Electrocoagulation Process. Ind. Eng. Chem. Res. 2008, 47 (6), 2018–2023. 10.1021/ie0714652.

[ref10] Zeng L.; Li X.; Liu J. Adsorptive Removal of Phosphate from Aqueous Solutions Using Iron Oxide Tailings. Water Res. 2004, 38 (5), 1318–1326. 10.1016/j.watres.2003.12.009. 14975665

[ref11] Vasudevan S.; Sozhan G.; Ravichandran S.; Jayaraj J.; Lakshmi J.; Sheela S. M. Studies on the Removal of Phosphate from Drinking Water by Electrocoagulation Process. Ind. Eng. Chem. Res. 2008, 47 (6), 2018–2023. 10.1021/ie0714652.

[ref12] Blaney L. M.; Cinar S.; SenGupta A. K. Hybrid Anion Exchanger for Trace Phosphate Removal from Water and Wastewater. Water Res. 2007, 41 (7), 1603–1613. 10.1016/j.watres.2007.01.008. 17306856

[ref13] Loganathan P.; Vigneswaran S.; Kandasamy J.; Bolan N. S. Removal and Recovery of Phosphate From Water Using Sorption. Crit Rev. Environ. Sci. Technol. 2014, 44 (8), 847–907. 10.1080/10643389.2012.741311.

[ref14] Wen Z.; Zhang Y.; Dai C. Removal of Phosphate from Aqueous Solution Using Nanoscale Zerovalent Iron (NZVI). Colloids Surf. A Physicochem Eng. Asp 2014, 457 (1), 433–440. 10.1016/j.colsurfa.2014.06.017.

[ref15] Cheung K. C.; Venkitachalam T. H. Improving Phosphate Removal of Sand Infiltration System Using Alkaline Fly Ash. Chemosphere 2000, 41 (1–2), 243–249. 10.1016/S0045-6535(99)00417-8. 10819207

[ref16] Guan Q.; Hu X.; Wu D.; Shang X.; Ye C.; Kong H. Phosphate Removal in Marine Electrolytes by Zeolite Synthesized from Coal Fly Ash. Fuel 2009, 88 (9), 1643–1649. 10.1016/j.fuel.2009.02.031.

[ref17] Pengthamkeerati P.; Satapanajaru T.; Chularuengoaksorn P. Chemical Modification of Coal Fly Ash for the Removal of Phosphate from Aqueous Solution. Fuel 2008, 87 (12), 2469–2476. 10.1016/j.fuel.2008.03.013.

[ref18] Bowden L. I.; Jarvis A. P.; Younger P. L.; Johnson K. L. Phosphorus Removal from Waste Waters Using Basic Oxygen Steel Slag. Environ. Sci. Technol. 2009, 43 (7), 2476–2481. 10.1021/es801626d. 19452904

[ref19] Barca C.; Gérente C.; Meyer D.; Chazarenc F.; Andrès Y. Phosphate Removal from Synthetic and Real Wastewater Using Steel Slags Produced in Europe. Water Res. 2012, 46 (7), 2376–2384. 10.1016/j.watres.2012.02.012. 22374297

[ref20] Akay G.; Keskinler B.; Çakici A.; Danis U. Phosphate Removal from Water by Red Mud Using Crossflow Microfiltration. Water Res. 1998, 32 (3), 717–726. 10.1016/S0043-1354(97)00236-4.

[ref21] Biswas B. K.; Inoue K.; Ghimire K. N.; Harada H.; Ohto K.; Kawakita H. Removal and Recovery of Phosphorus from Water by Means of Adsorption onto Orange Waste Gel Loaded with Zirconium. Bioresour. Technol. 2008, 99 (18), 8685–8690. 10.1016/j.biortech.2008.04.015. 18524574

[ref22] Liu H.; Sun X.; Yin C.; Hu C. Removal of Phosphate by Mesoporous ZrO2. J. Hazard Mater. 2008, 151 (2–3), 616–622. 10.1016/j.jhazmat.2007.06.033. 17658689

[ref23] Namasivayam C.; Prathap K. Recycling Fe(III)/Cr(III) Hydroxide, an Industrial Solid Waste for the Removal of Phosphate from Water. J. Hazard Mater. 2005, 123 (1–3), 127–134. 10.1016/j.jhazmat.2005.03.037. 15955623

[ref24] Zhang G.; Liu H.; Liu R.; Qu J. Removal of Phosphate from Water by a Fe-Mn Binary Oxide Adsorbent. J. Colloid Interface Sci. 2009, 335 (2), 168–174. 10.1016/j.jcis.2009.03.019. 19406416

[ref25] Lee Y.; Zimmermann S. G.; Kieu A. T.; Von Gunten U. Ferrate (Fe(VI)) Application for Municipal Wastewater Treatment: A Novel Process for Simultaneous Micropollutant Oxidation and Phosphate Removal. Environ. Sci. Technol. 2009, 43 (10), 3831–3838. 10.1021/es803588k. 19544895

[ref26] Wen Z.; Zhang Y.; Dai C. Removal of Phosphate from Aqueous Solution Using Nanoscale Zerovalent Iron (NZVI). Colloids Surf. A Physicochem Eng. Asp 2014, 457 (1), 433–440. 10.1016/j.colsurfa.2014.06.017.

[ref27] Acelas N. Y.; Martin B. D.; López D.; Jefferson B. Selective Removal of Phosphate from Wastewater Using Hydrated Metal Oxides Dispersed within Anionic Exchange Media. Chemosphere 2015, 119, 1353–1360. 10.1016/j.chemosphere.2014.02.024. 24630462

[ref28] Li G.; Gao S.; Zhang G.; Zhang X. Enhanced Adsorption of Phosphate from Aqueous Solution by Nanostructured Iron(III)–Copper(II) Binary Oxides. Chemical Engineering Journal 2014, 235, 124–131. 10.1016/j.cej.2013.09.021.

[ref29] Du Y.; Wang X.; Nie G.; Xu L.; Hu Y. Enhanced Phosphate Removal by Using La-Zr Binary Metal Oxide Nanoparticles Confined in Millimeter-Sized Anion Exchanger. J. Colloid Interface Sci. 2020, 580, 234–244. 10.1016/j.jcis.2020.07.011. 32683120

[ref30] Moumen E.; Bazzi L.; El Hankari S. Metal-Organic Frameworks and Their Composites for the Adsorption and Sensing of Phosphate. Coord. Chem. Rev. 2022, 455, 214376 10.1016/j.ccr.2021.214376.

[ref31] Maiti S.; Pramanik A.; Mahanty S. Extraordinarily High Pseudocapacitance of Metal Organic Framework Derived Nanostructured Cerium Oxide. Chem. Commun. 2014, 50 (79), 11717–11720. 10.1039/C4CC05363J. 25143153

[ref32] Liu K.; You H.; Jia G.; Zheng Y.; Huang Y.; Song Y.; Yang M.; Zhang L.; Zhang H. Hierarchically Nanostructured Coordination Polymer: Facile and Rapid Fabrication and Tunable Morphologies. Cryst. Growth Des 2010, 10 (2), 790–797. 10.1021/cg901170j.

[ref33] Elhussein E. A. A.; Şahin S.; Bayazit Ş. S. Preparation of CeO_2_ nanofibers Derived from Ce-BTC Metal-Organic Frameworks and Its Application on Pesticide Adsorption. J. Mol. Liq. 2018, 255, 10 10.1016/j.molliq.2018.01.165.

[ref34] Xie A.; Dai J.; Chen X.; He J.; Chang Z.; Yan Y.; Li C. Hierarchical Porous Carbon Materials Derived from a Waste Paper Towel with Ultrafast and Ultrahigh Performance for Adsorption of Tetracycline. RSC Adv. 2016, 6 (77), 72985–72998. 10.1039/C6RA17286E.

[ref35] Michelsen O. B. Photometric Determination of Phosphorus as Molybdovanadophosphoric Acid. Anal. Chem. 1957, 29 (1), 60–62. 10.1021/ac60121a017.

[ref36] Lagergren S. Zur Theorie Der Sogenannten Adsorption Geloster Stoffe. K. Sven. Vetenskad. Handl. 1898, 24, 1–39.

[ref37] Ho Y. S.; McKay G. Pseudo-Second Order Model for Sorption Processes. Process Biochem. 1999, 34 (5), 451–465. 10.1016/S0032-9592(98)00112-5.

[ref38] Largitte L.; Pasquier R. A Review of the Kinetics Adsorption Models and Their Application to the Adsorption of Lead by an Activated Carbon. Chem. Eng. Res. Des. 2016, 109, 495–504. 10.1016/j.cherd.2016.02.006.

[ref39] Wu F. C.; Tseng R. L.; Juang R. S. Initial Behavior of Intraparticle Diffusion Model Used in the Description of Adsorption Kinetics. Chemical Engineering Journal 2009, 153 (1–3), 1–8. 10.1016/j.cej.2009.04.042.

[ref40] Weber W. J.; Morris J. C. Kinetics of Adsorption on Carbon from Solution. J. Santi. Eng. Div. ASCE 1963, 89, 31–59. 10.1061/JSEDAI.0000430.

[ref41] Langmuir I. THE ADSORPTION OF GASES ON PLANE SURFACES OF GLASS, MICA AND PLATINUM. J. Am. Chem. Soc. 1918, 40 (9), 1361–1403. 10.1021/ja02242a004.

[ref42] Freundlich H. Adsorption in Solids. Z. Phys. Chem. 1906, 57, 385–470. 10.1515/zpch-1907-5723.

[ref43] Temkin M. J.; Pyzhev V. Recent Modifications to Langmuir Isotherms. Acta Phys.-Chim. Sin. URSS 1940, 12, 217–225.

[ref44] Rajahmundry G. K.; Garlapati C.; Kumar P. S.; Alwi R. S.; Vo D.-V. N. Statistical Analysis of Adsorption Isotherm Models and Its Appropriate Selection. Chemosphere 2021, 276, 130176 10.1016/j.chemosphere.2021.130176. 33714156

[ref45] Dubinin M. M.; Radushkevich L. V. Equation of the Characteristic Curve of Activated Charcoal. Chem. Zentr. 1947, 1 (1), 875.

[ref46] Schneider C. A.; Rasband W. S.; Eliceiri K. W. NIH Image to ImageJ: 25 Years of Image Analysis. Nat. Methods 2012, 9 (7), 671–675. 10.1038/nmeth.2089. 22930834 PMC5554542

[ref47] Almasri D. A.; Saleh N. B.; Atieh M. A.; McKay G.; Ahzi S. Adsorption of Phosphate on Iron Oxide Doped Halloysite Nanotubes. Sci. Rep. 2019, 9 (1), 1–13. 10.1038/s41598-019-39035-2. 30824719 PMC6397243

[ref48] Chubar N. I.; Kanibolotskyy V. A.; Strelko V. V.; Gallios G. G.; Samanidou V. F.; Shaposhnikova T. O.; Milgrandt V. G.; Zhuravlev I. Z. Adsorption of Phosphate Ions on Novel Inorganic Ion Exchangers. Colloids Surf. A Physicochem Eng. Asp 2005, 255 (1–3), 55–63. 10.1016/j.colsurfa.2004.12.015.

[ref49] Dichiara A. B.; Sherwood T. J.; Rogers R. E. Binder Free Graphene-Single-Wall Carbon Nanotube Hybrid Papers for the Removal of Polyaromatic Compounds from Aqueous Systems. J. Mater. Chem. A Mater. 2013, 1 (46), 14480–14483. 10.1039/c3ta13968a.

[ref50] Hubbe M.; Azizian S.; Douven S. Implications of Apparent Pseudo-Second-Order Adsorption Kinetics onto Cellulosic Materials: A Review. Bioresources 2019, 14 (3), 7582–7626. 10.15376/biores.14.3.7582-7626.

[ref51] Ahmad A. L. L.; Chan C. Y. Y.; Abd Shukor S. R. R.; Mashitah M. D. D. Adsorption Kinetics and Thermodynamics of β-Carotene on Silica-Based Adsorbent. Chemical Engineering Journal 2009, 148 (2–3), 378–384. 10.1016/j.cej.2008.09.011.

[ref52] He J.; Xu Y.; Wang W.; Hu B.; Wang Z.; Yang X.; Wang Y.; Yang L. Ce(III) Nanocomposites by Partial Thermal Decomposition of Ce-MOF for Effective Phosphate Adsorption in a Wide PH Range. Chemical Engineering Journal 2020, 379, 122431 10.1016/j.cej.2019.122431.

[ref53] Mohammadi R.; Hezarjaribi M.; Ramasamy D. L.; Sillanpää M.; Pihlajamäki A. Application of a Novel Biochar Adsorbent and Membrane to the Selective Separation of Phosphate from Phosphate-Rich Wastewaters. Chemical Engineering Journal 2021, 407, 126494 10.1016/j.cej.2020.126494.

[ref54] Gao Y.; Li Y.; Zhang L.; Huang H.; Hu J.; Shah S. M.; Su X. Adsorption and Removal of Tetracycline Antibiotics from Aqueous Solution by Graphene Oxide. J. Colloid Interface Sci. 2012, 368 (1), 540–546. 10.1016/j.jcis.2011.11.015. 22138269

[ref55] Hu Q.; Zhang Z. Application of Dubinin–Radushkevich Isotherm Model at the Solid/Solution Interface: A Theoretical Analysis. J. Mol. Liq. 2019, 277, 646–648. 10.1016/j.molliq.2019.01.005.

[ref56] Wu B.; Wan J.; Zhang Y.; Pan B.; Lo I. M. C. Selective Phosphate Removal from Water and Wastewater Using Sorption: Process Fundamentals and Removal Mechanisms. Environ. Sci. Technol. 2020, 54, 50 10.1021/ACS.EST.9B05569. 31804806

[ref57] Hassan M. H.; Stanton R.; Secora J.; Trivedi D. J.; Andreescu S. Ultrafast Removal of Phosphate from Eutrophic Waters Using a Cerium-Based Metal-Organic Framework. ACS Appl. Mater. Interfaces 2020, 12 (47), 52788–52796. 10.1021/ACSAMI.0C16477. 33198461

[ref58] Momma K.; Izumi F. VESTA 3 for Three-Dimensional Visualization of Crystal, Volumetric and Morphology Data. J. Appl. Crystallogr. 2011, 44 (6), 1272–1276. 10.1107/S0021889811038970.

[ref59] Suresh Kumar P.; Korving L.; Keesman K. J.; van Loosdrecht M. C. M.; Witkamp G. J. Effect of Pore Size Distribution and Particle Size of Porous Metal Oxides on Phosphate Adsorption Capacity and Kinetics. Chemical Engineering Journal 2019, 358, 160–169. 10.1016/j.cej.2018.09.202.

[ref60] Deng L.; Shi Z. Synthesis and Characterization of a Novel Mg–Al Hydrotalcite-Loaded Kaolin Clay and Its Adsorption Properties for Phosphate in Aqueous Solution. J. Alloys Compd. 2015, 637, 188–196. 10.1016/j.jallcom.2015.03.022.

[ref61] Fan T.; Wang M.; Wang X.; Chen Y.; Wang S.; Zhan H.; Chen X.; Lu A.; Zha S. Experimental Study of the Adsorption of Nitrogen and Phosphorus by Natural Clay Minerals. Adsorpt. Sci. Technol. 2021, 2021, 1 10.1155/2021/4158151.

[ref62] Sudhakaran S.; Mahadevan H.; Fathima S. L.; Krishnan K. A. Performance of Novel Pillared Eggshell-Bentonite Clay Bio-Composite for Enhanced Phosphate Adsorption from Aqueous Media. Groundwater Sustainable Dev. 2023, 22, 100960 10.1016/J.GSD.2023.100960.

[ref63] Adil S.; Kim J. O. The Effectiveness and Adsorption Mechanism of Iron-Carbon Nanotube Composites for Removing Phosphate from Aqueous Environments. Chemosphere 2023, 313, 137629 10.1016/j.chemosphere.2022.137629. 36565757

[ref64] Lu X.; Guo W.; Wang B.; Feng Y.; He S.; Xue L. Screening Optimal Preparation Conditions of Low-Cost Metal-Modified Biochar for Phosphate Adsorption and Unraveling Their Influence on Adsorption Performance. J. Clean Prod 2023, 425, 138927 10.1016/j.jclepro.2023.138927.

[ref65] Banu H. A. T.; Karthikeyan P.; Vigneshwaran S.; Meenakshi S. Adsorptive Performance of Lanthanum Encapsulated Biopolymer Chitosan-Kaolin Clay Hybrid Composite for the Recovery of Nitrate and Phosphate from Water. Int. J. Biol. Macromol. 2020, 154, 188–197. 10.1016/j.ijbiomac.2020.03.074. 32171829

[ref66] Shang Y.; Guo K.; Jiang P.; Xu X.; Gao B. Adsorption of Phosphate by the Cellulose-Based Biomaterial and Its Sustained Release of Laden Phosphate in Aqueous Solution and Soil. Int. J. Biol. Macromol. 2018, 109, 524–534. 10.1016/j.ijbiomac.2017.12.118. 29275199

[ref67] Manyatshe A.; Cele Z. E. D.; Balogun M. O.; Nkambule T. T. I.; Msagati T. A. M. Chitosan Modified Sugarcane Bagasse Biochar for the Adsorption of Inorganic Phosphate Ions from Aqueous Solution. J. Environ. Chem. Eng. 2022, 10 (5), 108243 10.1016/j.jece.2022.108243.

[ref68] Chen N.; Hu W.; Feng C.; Zhang Z. Removal of Phosphorus from Water Using Scallop Shell Synthesized Ceramic Biomaterials. Environ. Earth Sci. 2014, 71 (5), 2133–2142. 10.1007/s12665-013-2618-2.

